# Uncommon Presentation of Giant Tophaceous Gout in the Hand: A Case Report

**DOI:** 10.2174/0115733971374467250522093438

**Published:** 2025-05-23

**Authors:** Hassan Zmerly, Ibrahim Akkawi, Damiano Alfio Ruinato, Manuela Moscato, Maurizio Draghetti, Francesco Pegreffi

**Affiliations:** 1 Orthopaedics and Traumatology Unit, Villa Erbosa Hospital, Bologna, Italy;; 2 Department of Medical and Surgical Sciences, University of Bologna, Bologna, Italy;; 3 Department of Medicine and Surgery, School of Medicine and Surgery, Kore University, Enna, Italy

**Keywords:** Gout, tophaceous mass, nodule excision, interphalangeal joint, pharmacological therapy, multi disciplinary approach

## Abstract

**Background:**

Tophaceous gout masses are characterized by the accumulation of monosodium urate crystals in peripheral joints and soft tissues. The most commonly involved areas are the metatarsophalangeal and knee joints. Finger/hand localization is uncommon. If not correctly treated, a finger tophaceous mass can grow and, in rare cases, reach an abnormally large size, termed “giant.”

**Aim:**

The aim of our study is to present a rare case of a large tophaceous mass of the hand, localized in the fourth finger, and to highlight the role of surgical excision combined with a multidisciplinary team approach.

**Case Report:**

We present a rare case of an 82-year-old woman affected by giant tophaceous gout in the left hand, localized to the extensor region of the proximal interphalangeal joint of the fourth finger. Clinical evaluation, MRI, and ultrasound imaging showed a 35 x 30 mm nodule in the soft tissue.

The lesion was successfully treated by mass excision and debridement of the extensor tendon. Histopathologic examination confirmed the diagnosis of tophaceous gout. Post-operatively, a combination of medical and nutritional therapy was given. At a 3-year follow-up, the patient was free of symptoms with no evidence of disease in the fourth finger.

**Conclusion:**

Management of giant tophaceous gout in hand necessitates extensive mass excision combined with pharmacological therapy, dietary adjustments, and lifestyle modifications. Effective treatment of such cases requires a multidisciplinary team approach to address the complexity of the condition comprehensively.

## 1 INTRODUCTION

Gout is an inflammatory arthritis caused by an excess of uric acid in the blood. The progression of the disease leads to a secondary deposition of sodium monourate in the peripheral joints and soft tissues with the formation of tophi [1-3]. Depending on their location, tophi growth can lead to permanent damage of joints and soft tissues, as well as internal organs, such as the kidneys [4]. Proper management is essential in order to avoid the development of tophaceous masses, which are commonly present in patients of advanced age [3-5]. Long-term gout management consists of personal lifestyle modifications, patient education, a low-purine diet, treatment of comorbidities, and continuous urate-lowering drug prescription at a dose that achieves monosodium urate crystal dissolution, using a treat-to-serum urate target approach [6-8]. This management strategy prevents gout flares and leads to regression of tophaceous nodules [9, 10]. While tophaceous gout can appear in every area of the body, hand/finger involvement with urate crystal deposition is not common and is generally associated with an advanced disease stage [11]. Mallinson *et al*. [12] reported 16.9% hand localization and 8.1% in the interphalangeal joints. However, large or giant mass sizes are rare [13-15]. In the case of hand localization with inadequate medical treatment, large masses may cause cartilage fissuration and joint deformities and destruction, with possible ulcerations of the overlying skin which increases the risk of infection [16, 17].

The effective management of advanced gout stages with large masses involves several challenges; it implies mass excision combined with medical treatment. However, there is a lack of research in the literature that supports such management, the majority of published papers are case reports or small case number studies [14-19].

We present a rare case report of an 82-year-old woman affected by a large tophaceous gout mass in the left ring finger, successfully treated by mass excision combined with pharmacological therapy and dietary adjustments.

## 2 CASE REPORT

### 2.1 History

The patient was first referred to our Musculo Skeletal outpatient department, with a history of multiple yellow-white painless subcutaneous nodules in both hands and feet, with a painful, large mass in the fourth left hand finger. The patient was also affected by diabetes, hypertension, and gout. No kidney diseases related to gout were present. Hyperuricaemia was first diagnosed at the age of 55, and the dietary and medical treatment for hyperuricaemia was administered consisting of allopurinol 100 mg/day.

About 5 years later, however, she became non-compliant with both the medical and dietary treatments. Her general practitioner had been prescribing colchicine and Nonsteroidal Anti-Inflammatory Drugs (NSAIDs) on an occasional basis for the previous 10 years. The patient reported having no family history of gout.

### 2.2 Physical Examination

**Fig. (1):**
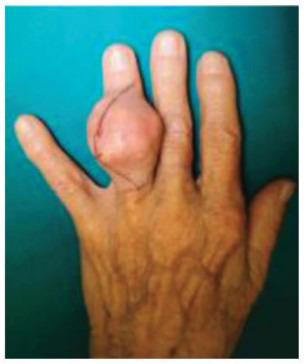
Tophaceous large mass located in the dorsal region of the left hand.

The patient presented with a painful, large mass in the dorsal region of the left hand at the level of the extensor side of the proximal interphalangeal joint of the fourth finger. The mass 35x30 mm in diameter (Fig. **1**) was causing deformity of the proximal interphalangeal joint and severely limiting the range of motion in terms of both active and passive flexion/extension. The patient was also noted to have less prominent, palpable, non-tender formations in the hands and feet.

### 2.3 Investigation

The laboratory examination showed serum uric acid levels of 7.44 mg/dL, C-reactive protein (CRP) level of 3.5 mg/dL (normal < 0.5), creatinine level of 0.97 mg/dL (normal 0.6-1.2), urea level of 35 mg/dL (normal 10-40), and an eGFR within the normal range at 76.

Ultrasound and MRI imaging of the left hand revealed a nodular mass of approximately 35x30 mm with calcifications adhering to the surface of the extensor tendon of the fourth finger. The mass presented with low-to-intermediate signal intensity on T1-weighted images and high signal intensity on T2-weighted images.

### 2.4 Treatment

**Fig. (2):**
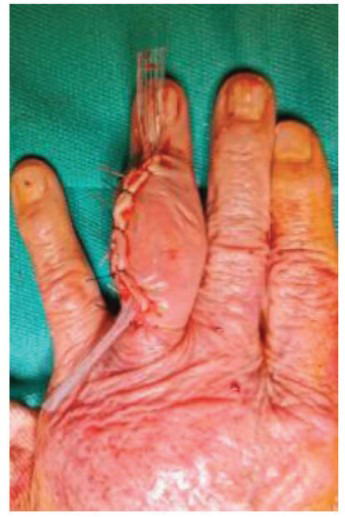
Enlarged mass excision *via* a dorsal approach.

The patient underwent mass excision using in-plexus anesthesia, with prior informed consent. Ceftriaxone was administered for antibiotic prophylaxis. The enlarged mass was excised through a dorsal incision (Fig. **2**), with collateral nerves isolated and protected. Debridement of the extensor tendons, which had been infiltrated by gout deposition, was also performed. Efforts were made to preserve tendon integrity as much as possible.

**Fig. (3):**
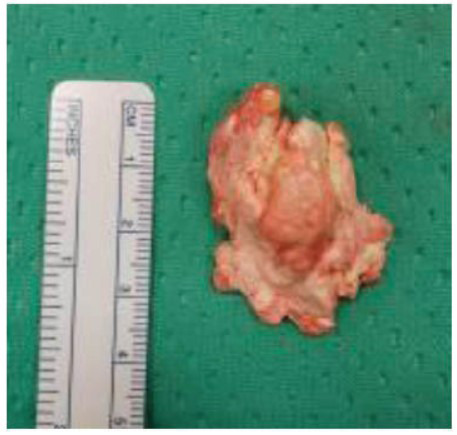
The macroscopic examination reveals a large nodule containing multiple small, white-speckled deposits.

Macroscopic examination revealed a large nodule with multiple small, white-speckled deposits (Fig. **3**). Histopathological examination confirmed the diagnosis of tophaceous gout.

Post-operatively, the patient experienced delayed wound healing for one month, which was managed with conservative treatment. The patient was followed by the rheumatology division and prescribed a course of allopurinol 300 mg, along with diet and lifestyle modifications.

### 2.5 Follow-up

At the 3- and 12-month follow-ups, the patient showed significant improvement in functionality and quality of life. At the 3-year follow-up, the patient had no active gout and had regained full flexion of the interphalangeal joint, although there was a sustained 10-degree loss in active extension. The patient reported complete satisfaction with her health outcome.

## 3 DISCUSSION

Gout is a metabolic disease caused by hyperuricaemia (serum urate > 6.8 mg/dL), leading to the precipitation of monosodium urate crystals inside and around the joints. This can result in acute, recurrent, or chronic arthritis [1-4]. Gouty arthritis is a common form of inflammatory arthritis that causes pain and swelling in the joints [17-19]. It typically lasts 1-2 weeks before resolving spontaneously.

The disease progresses through four phases: the early asymptomatic period, acute gouty arthritis, the intercritical phase, and chronic gout, which is characterized by needle- like monosodium urate crystal deposition and the development of tophaceous nodules [15, 20]. If hyperuricaemia remains untreated for a prolonged period, tophaceous nodules may form, typically in patients who have experienced hyperuricaemia for several years [21].

Tophaceous nodules usually appear in the metatarsophalangeal or knee joints but can present in any part of the musculoskeletal system [22-24].

A case series by Espinel *et al*. [18] reported that pain was the most common symptom (83%), followed by restricted range of motion (56%), deformity (50%), and difficulty with daily activities or work-related activities (28%).

Hung *et al*. [25] reported a case of extensor tendon rupture caused by gout disease. Nerve entrapment in the carpal canal and distal cubital syndrome were also reported [26-28].

In our case, the patient initially presented to us with a painful mass with deformity of the proximal interphalangeal joint and severe range of motion limitation.

Iwamoto *et al*. [29] also reported a case of an extensor tophaceous mass of the ring finger associated with painless, subcutaneous masses on her right hand and spontaneous loss of extension. It was concluded that given the spontaneous loss of extension, and in the case of chronic extensor tenosynovitis, the differential diagnosis must include gout disease of the hand.

Diagnostic investigation includes history, physical examination, plain radiography, ultrasound, computed tomography, and magnetic resonance imaging, which shows structural changes related to disease progression [24-26, 30].

The medical management of gout varies according to the clinical phase of the disease. Pharmacological therapies, such as Nonsteroidal Anti-inflammatory Drugs (NSAIDs), colchicine, and corticosteroids, remain the mainstay of symptomatic treatment during acute arthritis. On the other hand, long-term treatment is generally managed with dietary regimens and drugs that reduce uric acid levels, including xanthine oxidase inhibitors, which help prevent urate production, and uricosuric drugs, which facilitate urate excretion [22, 23]. In addition, interleukin-1 inhibitors (canakinumab, rilonacept, and anakinra) have recently been put to use as immunomodulators in order to reduce the inflammatory response. They can be administered as single or repeated doses [31].

Surgical treatment is rarely necessary [32]; however, in cases involving a giant nodule in the finger with the potential for severe complications, excision of the enlarged mass becomes essential [32]. In his study on the surgical management of gout, Zhang *et al*. [33] concluded that surgical intervention is a promising and safe therapeutic option. Nonetheless, dietary changes to reduce high purine intake and proper medical management of chronic hyperuricaemia remain the cornerstone treatments to prevent nodule formation and disease progression [16, 19, 24].

## CONCLUSION

Tophaceous nodules typically develop in patients with long-standing hyperuricaemia. If left untreated, these nodules can grow and reach large sizes. Giant tophaceous gout in the hand is a rare condition that requires prompt diagnosis and management with medical treatment. For large masses that are unresponsive to medical therapy, excision of the mass, in conjunction with medical treatment, dietary modifications, and lifestyle changes, is necessary. Mass excision can help prevent disease progression and joint destruction in cases of refractory gout.

Complete management of giant gout masses requires an interdisciplinary approach. Furthermore, careful follow-up is essential to prevent recurrence and complications.

## AUTHORS’ CONTRIBUTIONS

The authors confirm their contributions to the paper as follows: Study conception and design: HZ; methodology: MM; validation: MD and FP; draft manuscript: IA and DAR. All authors reviewed the results and approved the final version of the manuscript.

## Data Availability

All data generated or analyzed during this study are included in this published article.

## STANDARDS OF REPORTING

CARE guidelines were followed.
